# Systemic sclerosis complicating endometrial adenocarcinoma: A case report

**DOI:** 10.1097/MD.0000000000042553

**Published:** 2025-05-16

**Authors:** Hui-Chang Yan, Cui Huang

**Affiliations:** a Department of Dermatology, Henan General Hospital, Zhengzhou, Henan Province, China.

**Keywords:** endometrial adenocarcinoma, malignancy, pathogenesis, scleroderma, systemic sclerosis

## Abstract

**Rationale:**

Systemic sclerosis (SSc) is a rare systemic autoimmune disease, and it is even more uncommon to encounter it alongside a neoplasm.

**Patient concerns:**

A 55-year-old female patient presented to our clinic with a 7-year history of cyanosis affecting multiple fingers bilaterally, accompanied by Raynaud’s phenomenon, mild pain, numbness, and morning stiffness. One and a half years ago, she was diagnosed with endometrial adenocarcinoma (Federation International of Gynecology and Obstetrics stage IIIa) for frequent abdominal pain and abnormal vaginal bleeding.

**Diagnoses:**

Following a comprehensive physical examination and laboratory tests, she was diagnosed with SSc according to the 2013 American College of Rheumatology/European League Against Rheumatism classification criteria.

**Interventions:**

Although we discussed potential treatment options and prognosis with her, she ultimately declined therapy.

**Outcomes:**

She was lost to follow-up.

**Lessons:**

A shared pathophysiological process may underlie the development of both SSc and malignancy, possibly driven by persistent chronic inflammation and immune dysregulation.

## 1. Introduction

Systemic sclerosis (SSc) is an uncommon chronic multisystemic autoimmune disease characterized by dysregulated autoimmunity, microvasculopathy, and fibrosis of skin and visceral organs, with hitherto elusive etiology.

Though rarer, there are some reports of SSc with concomitant neoplasm, even accounting for some mortality. Solid organ cancers involving the lung (most common), breast, colon-rectum, liver, bladder, kidney, pancreas, esophagus, tongue, skin, cervix, etc, and hematological cancers (lymphoma, leukemia, multiple myeloma, and myeloproliferative disorders) have been reported to be concomitant.^[1,2]^ Although some instances of SSc occurring alongside various malignancies have been documented, the underlying mechanisms linking the 2 remain poorly understood. This underscores a significant research gap in exploring the shared pathophysiological pathways, genetic and environmental influences, and effective clinical management strategies for patients presenting with both conditions. Here, we present a rare case of SSc accompanied by endometrial adenocarcinoma.

## 2. Case report

**Figure 1.**
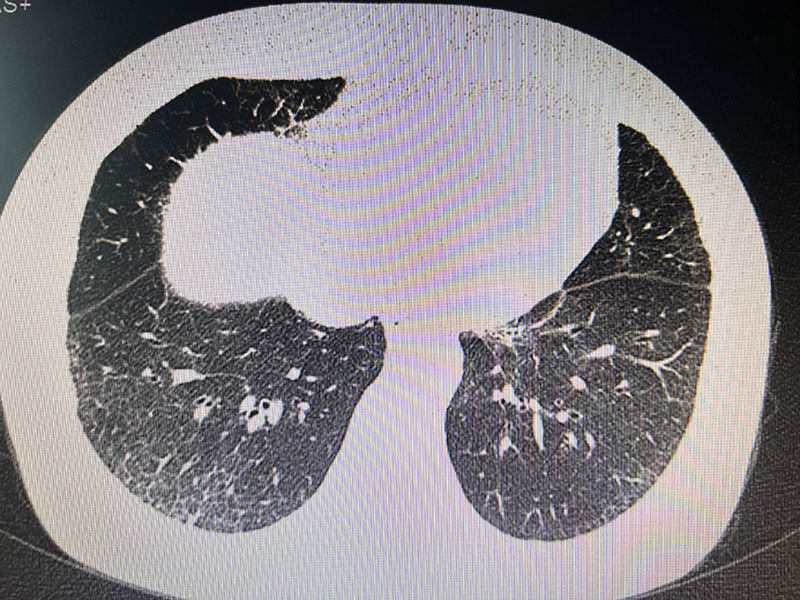
Computed tomography found some interstitial changes in the lower lobes of both lungs.

A 55-year-old female visited our clinic with a 7-year history of cyanosis of multiple fingers in both hands on March 16, 2023. Initially, cyanosis emerged after grasping the snowball, with various accompanying symptoms, such as the Raynaud phenomenon, mild pain, numbness, and morning stiffness, which may resolve partially after exercise. Occasional left knee ache, oral dryness, eye dryness, and muscle pain were also mentioned, without oral ulcer, alopecia, photosensitivity, or fatigue. Lesions progressively involved more fingers and crept to the proximal part in the last 2 years. One and a half years ago, frequent abdominal pain and abnormal vagina hemorrhages led her to visit a gynecologist, and she was diagnosed with endometrial adenocarcinoma (Federation International of Gynecology and Obstetrics staging IIIa) after a comprehensive screening. At the same time, some interstitial changes in the lower lobes of both lungs (Fig. 1) were also found simultaneously but overlooked. She underwent total abdominal hysterectomy, bilateral salpingo-oophorectomy, omentectomy, and pelvic lymph node dissection first, followed by 4 courses of chemotherapy with the scheme of paclitaxel and paraplatin and 2 classes of radiotherapy. During follow-up, no recurrence was found. Besides a 10-year history of type 2 diabetes mellitus, treated with dimethylbiguanide but with inadequate glycemic control, there were no other comorbidities.

**Figure 2.**
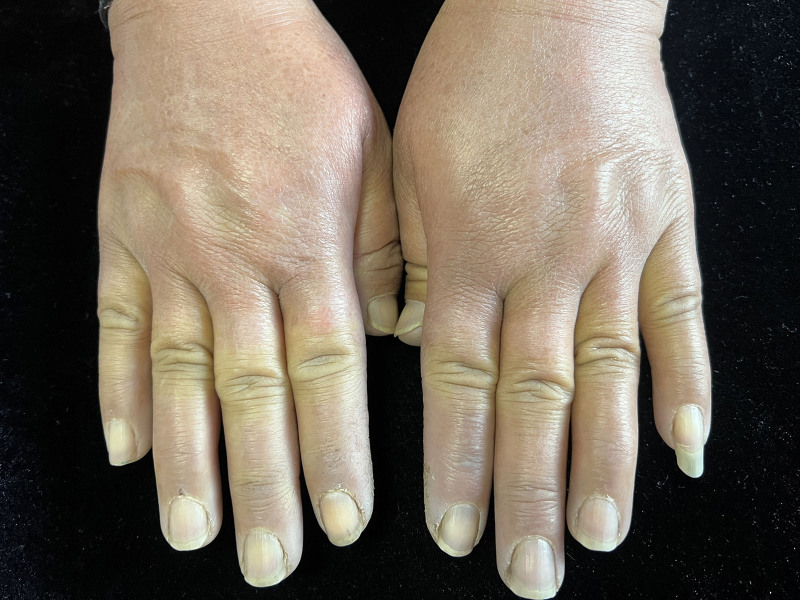
Cyanosis and mild swell in all fingers.

**Figure 3.**
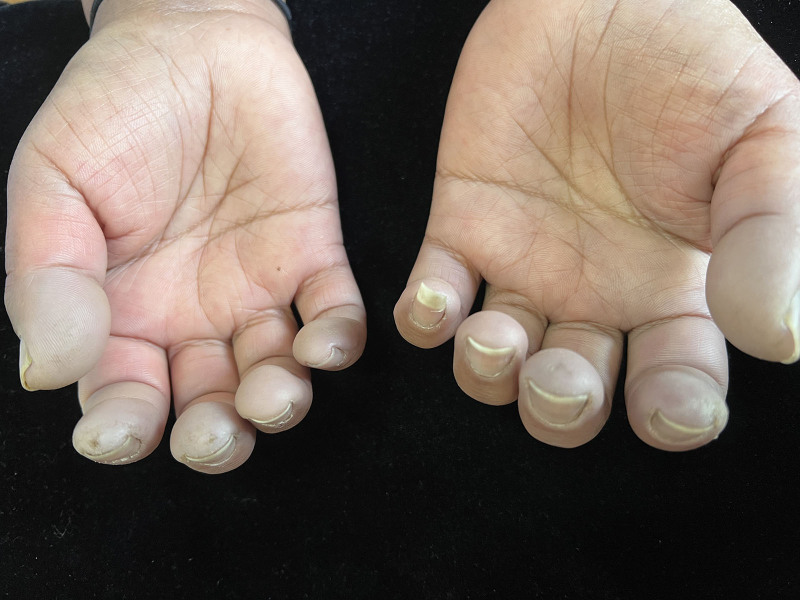
Some millet-sized atrophic scars are on the tip of both index finger and right middle finger.

Physical examination revealed cyanosis and mild swell involving all fingers with lowered skin temperature and moderate tenderness (Fig. 2), some millet size atrophic scar in the fingertip of both index finger and right middle finger (Fig. 3), diffuse hazel pigmentation in the face, and mild tenderness in the left knee.

According to her exceptional condition, we performed some primary screening examinations (Table 1). According to the 2013 American College of Rheumatology\European League Against Rheumatism criteria for the classification of SSc, the total score of our case was 13, which fulfilled the classification criteria (≥9), so a final diagnosis of SSc was made. However, she refused to receive therapy while she was informed of the following possible treatment and prognosis.

**Table 1:** Summary of results of screening examinations.

Examinations (reference value)	Results
Total white blood cell count ([4–10] × 10^9^/L)	3.34 × 10^9^/L
Urine glucose (negative)	1+
Fast blood glucose (3.89–6.11 mmol/L)	8.38 mmol/L
Anti-Scl-70 antibodies (negative)	(+)
Antinuclear antibody (negative)	(+), homogeneous pattern at titers 1:320
Rheumatoid factor (negative)	Negative
Antiphospholipid antibodies (negative)	Negative
Anti-neutrophil cytoplasm antibodies (negative)	Negative
Glutamic-pyruvic transaminase, glutamic oxaloacetic transaminase (0–40 U/L)	0
Complement 3 (0.9–1.5 g/L)	0.11
Complement 4 (0.2–0.4 g/L)	0.32
Erythrocyte sedimentation rate (0–20 mm/h)	15
C-reactive protein (0–5 mg/L)	1
Electrocardiogram	Normal
Computed tomography	Interstitial changes in both lower lung lobes

## 3. Discussion

SSc is a multisystem autoimmune disorder marked by microvascular dysfunction and immune abnormalities, leading to fibrosis of the skin and internal organs. Thick, hardened skin is the distinctive disease manifestation; hence, it has also been termed a “hidebound disease.” The skin over the extremities, face, and trunk may exhibit hyperpigmentation, with distinct depigmented regions. A “salt and pepper” appearance, characterized by vitiligo-like depigmentation and perifollicular hyperpigmentation, is a distinguishing skin manifestation. This typical appearance, together with skin sclerosis, is a diagnostic sign of SSc. Terminal phalanges resorption, short and claw-like fingers (due to acro-osteolysis), and fingertip ulcerations are also usually seen.^[3]^

A biphasic relevance between SSc and cancer has been demonstrated.^[1,2]^ While cancer occurs within the first 5 years of SSc onset, the pathological immune reaction in cancer and some anticancer therapies may trigger autoimmunity, resulting in SSc and supporting the hypothesis that SSc may be a paraneoplastic phenomenon. Otherwise, prolonged inflammation, persistent immunosuppressive conditions secondary to either treatment or altered immunity and acquired genetic functional defect of mesenchyma in SSc may predispose to cancerogenesis.^[1,2,4]^

A significant relevance between cancer and SSc implies that similar genetic settings and environmental risk factors are implicated in their pathogenesis.^[5]^ In addition, they share some similar pathophysiological mechanisms, namely, immune dysregulation, vasculogenesis, fibrosis, oxidative stress, etc.^[2]^

In 2011, Georgios Androutsopoulos and colleagues^[6]^ reported a 43-year-old female with SSc, endometrial cancer (stage Ib) endometrioid type, and ovarian cancer (stage IIIc) endometrioid type. To our knowledge, our case may be the 2nd case of SSc with cancers of the female reproductive system. Though the diagnosis of SSc was made 1 and a half years later than endometrial adenocarcinoma, the skin lesions occurred more than 5 years earlier, which suggests similar pathophysiological processes, such as persistent chronic inflammation, immunologic derangement, may be implicated in both pathogenesis.

In summary，our case supports the existence of fundamental pathophysiological links between SSc and oncogenesis, with chronic inflammatory persistence and breakdown of immune homeostasis emerging as plausible unifying mechanisms.

## Author contributions

**Conceptualization:** Hui-Chang Yan.

**Data curation:** Hui-Chang Yan, Cui Huang.

**Formal analysis:** Hui-Chang Yan.

**Investigation:** Cui Huang.

**Methodology:** Hui-Chang Yan.

**Project administration:** Hui-Chang Yan.

**Resources:** Hui-Chang Yan.

**Writing – original draft:** Hui-Chang Yan, Cui Huang.

**Writing – review & editing:** Hui-Chang Yan.
